# Evaluation of Diabetogenic Mechanism of High Fat Diet in Combination with Arsenic Exposure in Male Mice

**Published:** 2018

**Authors:** Akram Ahangarpour, Soheila Alboghobeish, Mohsen Rezaei, Mohammad Javad Khodayar, Ali Akbar Oroojan, Marzieh Zainvand

**Affiliations:** a *Health Research Institute, Diabetes Research Center, Department of Physiology, Ahvaz Jundishapur University of Medical Sciences, Ahvaz, Iran. *; b *Department of Pharmacology, School of Medicine, Student Research Committee of Ahvaz Jundishapur University of Medical Sciences, Ahvaz, Iran. *; c *Department of Toxicology, Faculty of Medical Sciences, Tarbiat Modares University, Tehran, Iran. *; d *Department of Pharmacology and Toxicology, School of Pharmacy, Jundishapur University of Medical Sciences, Ahvaz, Iran. *; e *Department of Physiology, Student Research Committee of Ahvaz Jundishapur University of Medical Science, Ahvaz, Iran.*; f *Department of Toxicology, School of Pharmacy, Student Research Committee of Ahvaz Jundishapur University of Medical Sciences, Ahvaz, Iran.*

**Keywords:** Type 2 diabetes, Arsenic, High-fat diet, Mitochondrial oxidative stress, Islet insulin secretion

## Abstract

Obesity is a main reason of type 2 diabetes and also chronic exposure to arsenic (As) can produce diabetic symptoms. In previous studies, the association between high-fat diet and arsenic in the incidence of diabetes was found, but the role of beta cells activity, liver mitochondrial oxidative stress, and hepatic enzymes (leptin, adiponectin and beta amylase) was unclear. Thus, present study was conducted to evaluate the diabetogenic mechanism of arsenic followed by concomitant administration of high-fat diet (HFD) in male mice. In this experimental study, the mice consumed with HFD or low-fat diet (LFD) while exposed to As 25 or 50 ppm in drinking water for 20 weeks. At the end of experiments, hyperglycemia, insulin resistance variables, lipid profile, hepatic enzymes, liver mitochondrial oxidative stress, islet insulin secretion, liver, and pancreas histopathology were evaluated in all mice by their own methods. Control HFD fed mice showed a significant increase in FBG, OGTT, HOMA-IR, ITT, lipid profile, leptin, β-amylase, liver mitochondrial oxidative stress, hepatic enzymes and decreased FPI, HOMA-β, adiponectin, and islet insulin secretion or content. However, exposure to HFD concomitant with Arsenic revealed an impressive reduction in FBG, FPI, HOMA-IR, HOMA-β, ITT, lipid profile, and islet insulin secretion or content. This exposure enhanced OGTT, leptin, adiponectin, liver mitochondrial oxidative stress, and hepatic enzymes. In conclusion, HFD and arsenic concomitant administration induced impairment of OGTT and islet insulin secretion or content through the mitochondrial oxidative stress.

## Introduction

Diabetes mellitus classified by type 1 (insulin-dependent diabetes mellitus) and type 2 (noninsulin-dependent diabetes mellitus) (1). Type 2 diabetes is a metabolic disorder distinguished by hyperglycemia, peripheral insulin resistance, and pancreatic β cells dysfunction. 

Further, impaired insulin secretion, decreased muscle glucose uptake, increased hepatic glucose production, and decreased hepatic glucose uptake have been occurred through the glucose intolerance in type 2 diabetic individuals. However, liver and muscle insulin resistance were established in this disease, but type 2 diabetes does not happen in the absence of progressive β cells damage. Impaired glucose tolerance (IGT) is performed near maximally insulin resistant in diabetic patients and they have approximately lost 80% of their β cells function (2). According to the World Health Organization’s (WHO) reports, the prevalence of this type of diabetes estimated about 346 million people worldwide and, accounts for 90%–95% of all diabetic cases (3). The main risk factors which attributed in type 2 diabetes are aging, obesity, physical inactivity, heredity, and oxidative stress. Moreover, environmental toxicants such as arsenic have been suggested as etiological factor in diabetes development (4).

Arsenic is distribute in Earth’s crust, and contaminates drinking water sources by leaching, erosion, and mining. High water’s concentration of arsenic has been reported in several countries in Asia, Europe and the Americas such as Iran, Bangladesh, China, India, Taiwan, Hungary, Argentina, Chile, Mexico, and USA. According to WHO reports about 140 million people are exposed to arsenic contaminated drinking water worldwide. This source of arsenic, as the main general population exposure, is more detrimental than arsenic in food because this form of arsenic is mainly inorganic with greater bio-availability (5). Although some studies revealed that exposure to high levels of arsenic decrease insulin secretion of pancreatic β cells and inhibits adipocytes glucose uptake that leads to insulin resistance, but it has yielded conflicting results for the relationship between type 2 diabetes and low or moderate concentration of arsenic in drinking water (6). Arsenic-induced oxidative stress may occur by interacting with antioxidant enzymes activity and results to the free radicals or reactive oxygen species (ROS) accumulation such as superoxide anion (O_2_^-^), hydroxyl radical (OH), and hydrogen peroxide (H_2_O_2_). Further, chronic administration of arsenic induced hepatotoxicity through the increase lipid peroxidation and GSH/GSSG ratio reduction and cause DNA damage in hepatocytes (7-9). Since pancreatic islets contains low amount of antioxidant enzymes, they are more sensitive to the damage of peroxide and ROS exposure (10). It has been reported that arsenic induced pathophysiological injury and β cell dysfunctions or apoptosis via induction of oxidative stress (11).

Mitochondria are intercellular energy production sources through the electron transport chain. Impairment cellular bioenergetics related diseases associated with mitochondrial oxidative stress. Arsenic increased ROS over generation and thiol oxidation by act as specific complex I directed inhibitors (12). Obesity as a significant health problem can cause to excess adipose tissue accumulation and energy homeostasis disturbance. This phenomenon known as a potentially preventable cause of premature morbidity and death, is associated with several disabilities (13). Epidemic prevalence of obesity is about 1.6 billion adults classified by overweight and obese (14). The epidemic consumption of a high-fat may results to a mark increase of type 2 diabetes outbreak (15). Previous study suggested that high fat diet (HFD)-induced obese is an appropriate model of obesity in rats (13).

As motioned above, the diabetogenic effects of HFD and arsenic have been revealed in the previous studies. But, the role of pancreatic β cells, liver’s mitochondria, leptin, adiponectin, and beta amylase has been unclear. Therefore, the aim of this study was conducted to evaluate the diabetogenic mechanism of arsenic followed by concomitant administration of HFD and, their effects on β cell’s insulin secretion and hepatic mitochondria in male mice.

## Experimental

*Chemicals*

Sodium arsenite (99% pure),4-(2-Hydroxyethyl)-1-piperazineethanesulfonic acid) (HEPES), mannitol, ethylene glycol tetra acetic acid (EGTA), bovine serum albumin (BSA), 2,7- dichlorofluoresceindiacetate

(DCFH-DA),3,43-(4,5-dimethylthiazol-2-yl)-2,5-iphenyltetrazolium bromide (MTT), Rhodamine 123, thiobarbituric acid, trichloroacetic acid ,1,1,3,3-tetramethoxypropane, reduced glutathione, oxidized glutathione, Coomassie Brilliant Blue were purchased from Sigma-Aldrich (St Louis, Missouri, USA), and sucrose 5, 5ꞌ-dithiobis-2-nitrobenzoic acid (DTNB) and dimethyl sulfoxide (DMSO) NaCl, KCl, CaCl_2_, MgCl_2_ and NaHCO_3_ were obtained from Merck company (Darmstadt, Germany). Collagenase type P was purchased from Roch Company (Germany). 

*Animal’s preparation*

Seventy-two adult male NMRI mice (30-35 g) were obtained from the animal facility of Ahvaz Jundishapur University of Medical Science (AJUMS), which is fully accredited by AJUMS animal care guidelines with an ethics committee grantee No. IR.AJUMS.REC.1394.604. The mice were housed six per cage in polycarbonate cages with corncob bedding in 20 ± 4 °C temperature with a 12 h light/12 h dark cycle and 10% humidity. The mice received a low-fat diet (LFD; 11% of all calorie supply from fat) or a high-fat diet (HFD; 57% of all calorie supply from fat) (purchased from Javaneh khorasan lab. Iran). According to some studies the grain-based diet contained 19.5–28.6 ppb arsenic (mainly iAs), and it may be compromised the training design. To evade this issue a purified diets without grain components has been used. The level of arsenic in high fat diet (58% Fat, 16.4% Protein, 25.5% Carbohydrate kcal/g)and low fat diet (11% Fat, 16% Protein, 72.8% Carbohydrate kcal/g)were 5 ppb and 7 ppb respectively that consists very low concentration of As compared to the administered concentration (25 and 50 ppm) in this study. The previous studies showed that mice might be less susceptible than human to arsenic toxicity, partly due to a faster metabolism and clearance of arsenic. Therefore, it is necessary to use higher exposure concentration of arsenic than the environmentally relevant concentrations in mouse experiment. Indeed, a recent report showed that it took 10 times higher concentration of drinking water arsenic (50 ppm) to achieve liver arsenic concentrations similar to those seen in human exposed to arsenic in west Bengal (16). Therefore, the mice drank diH_2_O or diH_2_O containing 25 or 50 ppm arsenic as arsenite in the present study. Water containing arsenite was freshly prepared every three days to minimize its oxidation. Water and food consumption, body weight monitoring, and fasting blood glucose (FBG) have been measured every week in all experimental groups. Therefore, the mice were divided into six groups (n = 12): LFD (control group), LFD + As 25 ppm, LFD + As 50 ppm, HFD (control group), HFD + As 25 ppm and, HFD + As 50 ppm (16).

*Oral glucose tolerance test (OGTT) and Insulin tolerance tests (ITT)*

After 20 experimental weeks, in order to OGTT assessment, all animals were fasted overnight and d-Glucose (2 g/kg of body weight; Sigma) dissolved in diH_2_O and orally gavaged to the fasted mice by a 20-gauge stainless steel gavage feeding needle (Fisher Scientific, Waltham). Then, blood samples (2 μL each) were immediately collected from a tail-clip bleed and blood glucose levels were measured using glucometer (Elegance CT-X10, Convergent Technologies, Germany) before and 30, 60, 90, and 120 min after glucose administration (16). Also, in order to ITT measurement, the mice were 12 h fasted after the experiments. Then, fasting blood glucose levels were measured before and 30, 60, 90, and 120 min after intraperitoneal injection of insulin (0.5 UI/kg body weight) (0.05 UI/mL; Insulin Humane injection USP, Vitasulin-R, Vitane Pharmed Co). Ultimately, Areas under the curve (AUC) for ITT were calculated to evaluate insulin resistance (17).

*Biochemical assessment*

Twenty-four hours after the last experimental day, the overnight fasting animals were anesthetized by ether. Fasting blood glucose was measured by cutting the tail tip and using glucometer (Elegance CT-X10, Convergent Technologies, Germany). Then, Blood samples were directly collected by cardiac puncture and centrifuged at 3500 rpm for 20 min. Plasma samples were stored at -70 °C until biochemical assessment were performed (18). Insulin level measurement was performed by ELISA assay kits (Monobind, USA) (The sensitivity of hormone detection per assay tube was 0.182 µIU/mL). Insulin resistance (HOMA-IR) and Homeostatic model assessment of pancreatic β-cell function (HOMA-β) were calculated according to following formula: 

HOMA-IR: fasting blood glucose (mg/dL) × insulin (µIU/mL)/405 (19).

HOMA-β: 20 × insulin (µIU/mL)/ (FBG (mmol/L) - 3.5) (20).

Plasma levels of total cholesterol (TC), triglyceride (TG), LDL-cholesterol (LDL-c), HDL-cholesterol (HDL-c), Alkaline phosphatase (ALP), Aspartate aminotransferase (AST), Alanine aminotransferase (ALT) and Tris-HCl (0.025M, pH 7.4) homogenized liver supernatant levels of triglyceride (TG), Alkaline phosphatase (ALP), Aspartate aminotransferase (AST), Alanine aminotransferase (ALT) were analyzed using commercial kits (Pars Azmoon, Iran) and, auto-analyzer method. The concentration of very low density lipoprotein cholesterol (VLDL –c) was calculated by Norbert formula (TG/5) (21). Also, the plasma leptin and adiponectin levels were evaluated by ELISA assay kits (Labor Diagnostika Nord GmbH, Germany) with low-end sensitivities of 0.5 ng/mL.

*Preparation of Mitochondria*

Mice liver’s mitochondria were isolated according to the Bermann and Hasting method (22). Briefly, rat’s liver was carefully removed, washed with buffer, and cut into small pieces. Liver pieces were homogenized in an ice-cold isolation buffer containing 1 mM EGTA, 215 mM mannitol, 75 mM sucrose, 0.1% BSA, and 20 mM HEPES. The tissue homogenate was centrifuged at 13,000×g for 5 min at 4 °C. In order to pellet the mitochondria, the micro tubes resuspended in 0.5 mL of isolation buffer and centrifuged again at 13,000×g for 10 min. Then, the pellets washed using appropriate buffer containing EGTA, spun at 13,000×g for 10 min, and resuspended in the buffer without EGTA.

*MTT assessment*

Isolated mitochondria viability was carried out using an MTT assay method. The containing mitochondria tubes were incubated at 37 °C for 1 h. After washing with the mitochondria suspension buffer, they were centrifuged at 1000×g for 20 min at 4 °C. Then, MTT solution (1 mL) was added to the each tube and incubated at 37 °C for 1 h. After centrifuging again at 1000×g for 20 min, DMSO (1 mL) was added to each tube and the tubes were shacked well. Then, 100 µL of each sample was poured in glass spectrophotometer cuvette, and the color intensity was measured by spectrophotometer reader at 570 nm. Ultimately, the mitochondrial viability was assessed as a percentage of control (23).

*Lipid peroxidation*

Mitochondrial lipid peroxidation was determined in terms of thiobarbituric acid reactive substances (TBARS) formation. A volume of 1 mL separated mitochondria solution was mixed with 250 µL trichloroacetic acid (70%) and centrifuged at 3000×g for 15 min. Then, 1 mL of supernatant was added to 1 mL TBA (0.8%) and heated at 100 °C for 30 min. The absorbance was read spectrophotometrically at 532 nm using a blank containing all the reagents except the tissue homogenate. The values were expressed as µg/mg protein. Since 99% of the TBARS was malondialdehyde (MDA), hence, TBARS concentrations of the samples were calculated from a standard curve using 1, 1, 3, 3-tetramethoxypropane (24).

*Determination of GSH and GSSG*

The mitochondrial samples were analyzed for reduced glutathione (GSH) by the 5, 5’-dithiobis-2-nitrobenzoic acid (DTNB) recycling procedure. DTNB (2 mL) was added to 1 mL of mitochondria sample for GSSG (oxidized glutathione) plus GSH determination, and the results were spectrophotometrically read at 412 nm using a blank containing 1 mL of isolation buffer and 2 mL of DTNB (25).

*Measurement of mitochondrial ROS*

The levels of ROS were measured by adding 1 mL of dichloro-dihydro-fluorescein diacetate (DCFH-DA) 3.32 M to 1 mL of isolated mitochondria sample. DCFH-DA penetrates into the mitochondria and hydrolyzes to non-fluorescent DCFH, trapped and oxidized to highly form of fluorescent 2, 7-dichlorofluorescein through the reaction with ROS. Then, fluorescence intensity was measured spectrofluorometerically (UV-1650PC SHIMADZU, Kyoto, Japan; Ex ¼ 500 nm, Em ¼ 520 nm) (26).

*MMP assessment*

The mitochondrial membrane potential (ΔΨm; MMP) collapse was measured using rhodamine 123 as a cationic fluorescent probe. Rhodamine 123 would accumulate more in healthier mitochondria matrices. Further, the red-to-green fluorescence ratio is decreased compared to healthy mitochondria upon mitochondrial damage. Fluorescence intensity was measured spectrofluorometerically (LS50B PerkinElmer, Waltham, Massachusetts, USA; Ex ¼ 490 nm, Em ¼ 535 nm) (26).

*Liver analysis for arsenic absorption*

Arsenic was determined by an atomic absorption spectrophotometer in the liver samples (Perkin-Elmer4100 Perkin Elmer Norwalk, Connecticut). Tissue samples (0.02-0.1 g, n = 7 per each group) were digested using 2 mol/L phosphoric acid (27). 

*Islet isolation*

The pancreas of animals were excised by a V-incision at the genital area and transferred into a petri dish containing 50 mL Krebs-bicarbonate buffer (115 mM NaCl, 5 mM KCl, 2.56 mM CaCl2, 1 mM MgCl2, 10 mM NaHCO3, 15 mM HEPES, supplemented with 0.5% bovine serum albumin (BSA) and balanced with a mixture of 95% oxygen, 5% carbon dioxide, pH 7.4) (28) and cut into (1 mm × 1 mm) pieces. The contents of petri dish centrifuged at 100×g for 5 min and, the obtained supernatant was removed and transferred to a 15 mL conical tube. In order to isolated islets purification from exocrine tissues, collagenase type P (Roch Company, Germany) (1-2 mg/pancreas) was added to the conical tube solution and inserted in a shaking water bath 800 oscillations shake for 5-10 min at 37 °C. Then, 15 mL of cold Krebs-bicarbonate buffer was added in conical tube solution to stop digestion and centrifuged at 500×g for 5 min. Supernatant of sample was aspirated and resuspended to a blackened petri dish. Ultimately, islets dissection was carried out manually using drawn-out glass pipette under stereomicroscope observation (29).

*Islet insulin secretion and content*

Isolated islets were transferred to 2 mL micro tubes containing Krebs-bicarbonate buffer with 5.6 mM of d-Glucose concentrations (similar to the fasting blood glucose) (30). The tubes were shaken using vortex and incubated at 37 °C for 45 min. After incubation, the samples were centrifuged at 100×g for 5 min and, 0.9 mL of sample’s supernatant maintained at −70 °C until the insulin secretion assay was performed. The same protocol was performed for insulin content assessment just HCL 0.18 M in ethanol 96% was added into the incubated solution after 30 min and the incubation period was again carried out for 24 h. Each Petri dish contains 10 islets and the *in-vitro* protocol repeated 8 times for each animal in all groups (31).

*Histopathological Assessment*

Pancreas and liver of animals were removed, fixed in 10% formalin solution, dehydrated in graded alcohol concentrations and, embedded in paraffin. Sections of 4-6 µm were prepared and stained with hematoxylin and eosin (H and E). Pancreas histopathology was assessed using light microscopy (Olympus PX 50 F3 model, Japan). Finally, a “blind” method has been used for slides reading (32).

*Statistical analysis*

Data were presented as means ± SE for three different experiments. All the results were analyzed using Graph Pad Prism (version 5.04). Statistical significance was determined using the one-way analysis of variance with the Tukey post-hoc test. Statistical significance was set at *p* < 0.05.

## Results

*Effect of diet and arsenic exposure on water, arsenic, food, and calorie intakes*

**Figure 1:**
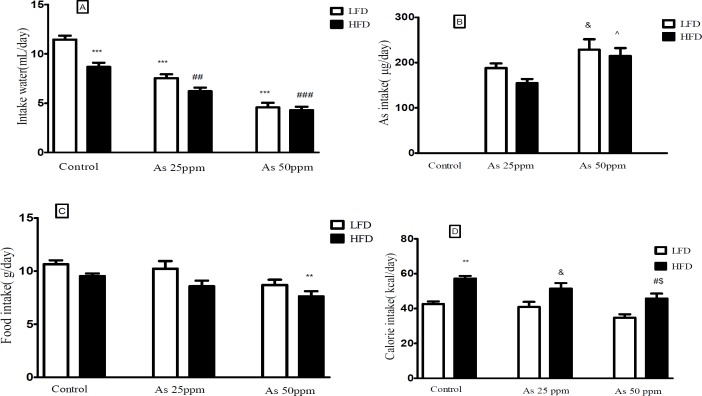
A) Intakes of water, (B) As, (C) food, and (D) calories by control LFD or HFD fed and As 25 or 50 treated LFD or HFD mice. Values represented as mean ± SD (n = 12, for A-D).*: Significantly different from LFD, #: Significantly different from HFD, &: Significantly different from LFD + As 25 ppm, ^: Significantly different from HFD + As 25 ppm, $: Significantly different from LFD + As 50 ppm. *, #, &, ^ and $ *p* < 0.05, ** and ## *p* < 0.01, *** and ### *p *< 0.001

Daily water, food, and calorie intakes were significantly affected by type of diet and arsenic exposure (Figure 1). Control mice consumed less water on HFD than LFD mice (8.68 and 11.46 mL/day respectively, *p* < 0.001). Water intake decreased in both arsenic concentration exposed animals when compared to their control groups. LFD mice exposed to As 25 and 50 ppm drank 7.53 and 4.61 mL/day respectively (*p* < 0.001) and HFD mice exposed to As 25 and 50 ppm drank 6.20 and 4.29 mL/day respectively (*p* < 0.01) (Figure 1A).

**Table 1:** Effect of high fat diet and arsenic on plasma lipid profiles and liver TG.

** Groups**	**Low fat diet**			**High fat diet**		
**Variables**						
TG	92.4 ± 3.1	73.2 ± 5.1	69.1 ± 3.3	121.5 ± 10.6*	87.7 ± 3.5#	63.4 ± 2.4#
Liver TG	53.6 ± 4.5	67.1 ± 9.3	79.5 ± 14.7	144.5 ± 25.8***	98.1 ± 13.6#	79.7 ± 13.7##
Cholesterol	93.1 ± 4.3	89.8 ± 6.7	101.5 ± 11.5	192.8 ± 13.9**	170.6 ± 11.6&&	158.8 ± 21.4#$
HDL	83.4 ± 6.8	80.5 ± 6.5	80.8 ± 4.9	113.1 ± 11.7**	117.5 ± 10.5&&	110.8 ± 8.4$$
LDL	13.8 ± 5.2	14.5 ± 3.6	16.1 ± 3.5	27.5 ± 3.4**	24.3 ± 4.2&	20.1 ± 7.5#
VLDL	18.5 ± 0.7	14.6 ± 1.0	13.8 ± 0.8	24.3 ± 2.2*	17.5 ± 0.7#	12.6 ± 0.6#

All data are given as Mean ± SEM of different experiments (n = 12). TG; triglyceride, HDL; high density lipoprotein, LDL; low density lipoprotein, VLDL; very low density lipoprotein. *: Significantly different from LFD, #: Significantly different from HFD, &: Significantly different from LFD + As 25 ppm, $: Significantly different from LFD + As50 ppm.

*, & and *p* < 0.05,

** , && and *p *< 0.01,

*** *p* < 0.001.

**Table 2:** Effect of high fat diet and arsenic on the diameter of islets, Values with different superscripts within the same column significantly differ from each other (*p <* 0.05).

Groups	**Low fat diet**			**High fat diet**		
Variables	**Control**	**As 25 ppm**	**As 50 ppm**	**Control**	**As 25 ppm**	**As 50 ppm**
Diameter of islets	394.5 ± 36.1	265.2 ± 25.1	107.4 ± 13.3	358.6 ± 31.7*	217.8 ± 23.5#	91.9 ± 9.4#

All data are given as Mean ± SEM of different experiments (n = 12).

* : Significantly different from LFD,

#: Significantly different from HFD,

* , # p < 0.05.

**Figure 2:**
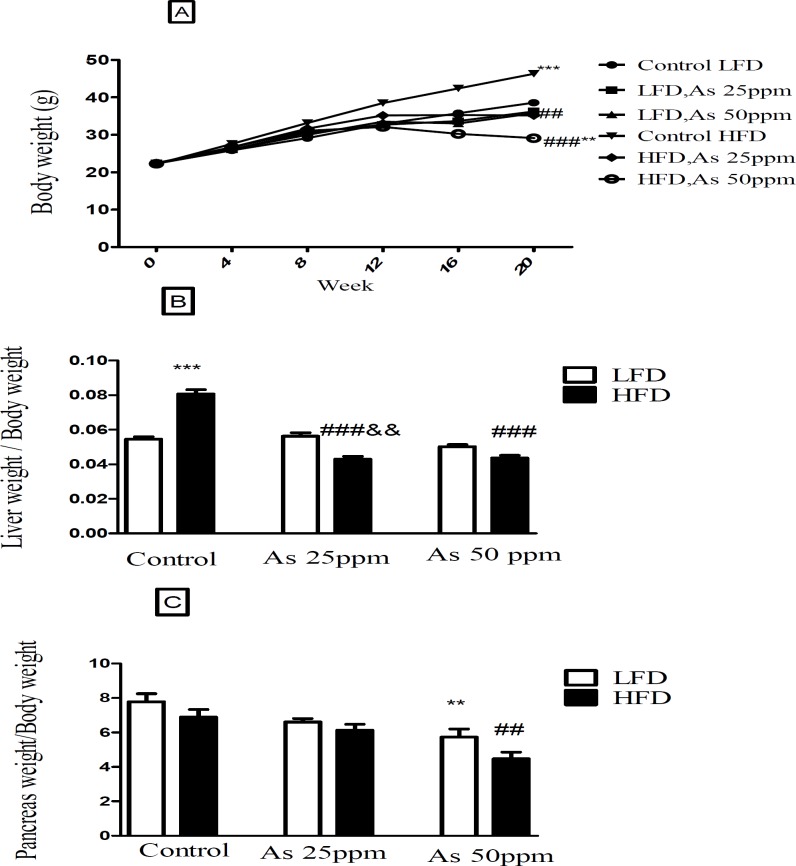
Bodyweight and liver and pancreas weight to body weight ratio in control LFD or HFD fed and As 25 or 50 treated LFD or HFD mice. (A) Body weight. (B) Liver weight to body weight ratio. (C) Pancreas weight to body weight ratio. Values represented as mean ± SD (n = 12, for A-C). *: Significantly different from LFD, #: Significantly different from HFD, &: Significantly different from LFD + As 25 ppm. **, ## and && *p* < 0.01, *** and ###, *p* < 0.001

**Figure 3:**
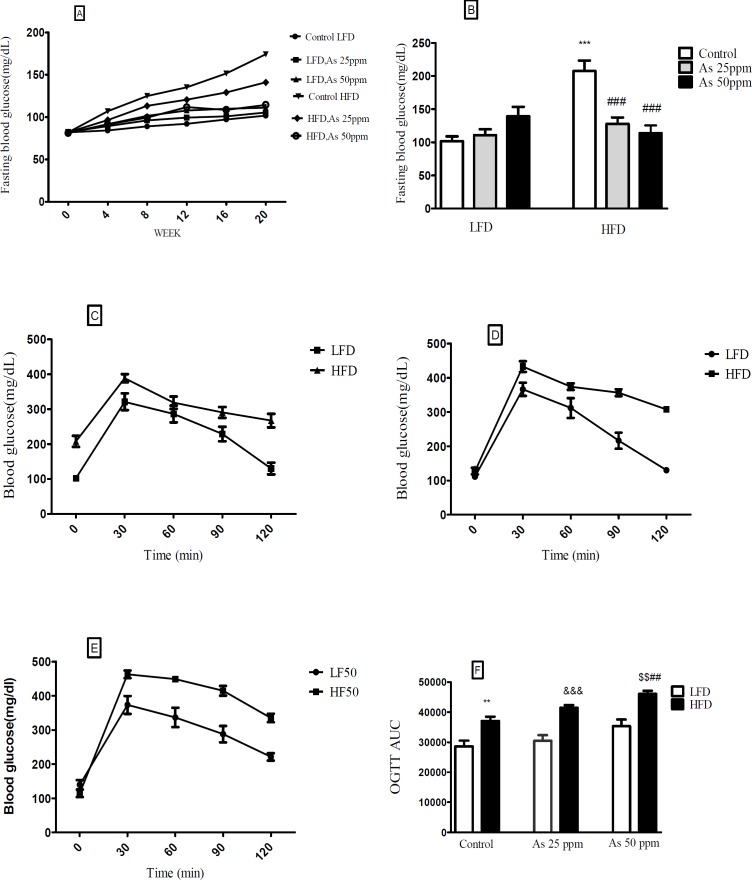
Effects of As exposure on blood glucose and OGTT in control LFD or HFD fed and As 25 or 50 treated LFD or HFD mice. (A) Fasting blood glucose; (B) Fasting blood glucose after 20 weeks (C) OGTT results for control mice (D) OGTT results for mice exposed to As 25 ppm (E) OGTT results for mice exposed to As 50 ppm (F) OGTT AUC, calculated according to OGTT. Values represented as mean ± SD (n = 12, for A-F). *: Significantly different from LFD, #: Significantly different from HFD, &: Significantly different from LFD + As 25 ppm, $: Significantly different from LFD + As50 ppm. **, ## and $$ *p* < 0.01, ***, ### and &&& *p* < 0.001

**Figure 4:**
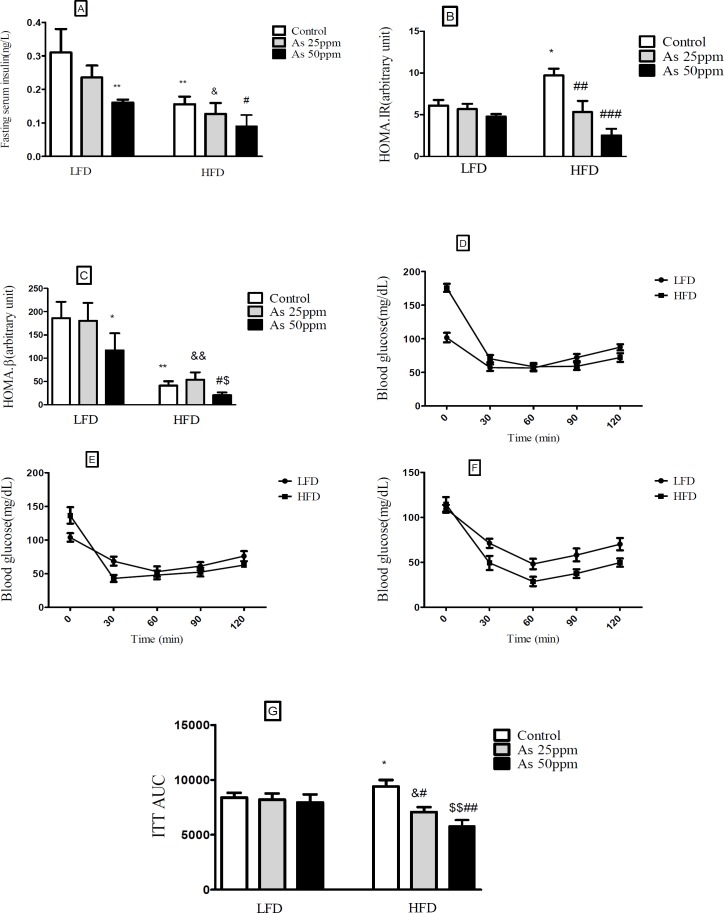
Effects of As exposure on glucose and insulin tolerance in control LFD or HFD fed and As 25 or 50 treated LFD or HFD mice (A) Fasting plasma insulin; (B) HOMA-IR; (C) HOMA-β; (D) ITT results for control mice; (E) ITT results for mice exposed to As 25 ppm; (F) ITT results for mice exposed to As 50 ppm (G) ITT AUC, calculated according to ITT calculated. Values represented as mean ± SD (n = 12, for A-G). *: Significantly different from LFD, #: Significantly different from HFD, &: Significantly different from LFD + As 25 ppm, $: Significantly different from LFD + As 50 ppm. * #, & and $ *p* < 0.05, **, ##, && and $$ *p* < 0.01, ### *p* < 0.001

**Figure 5:**
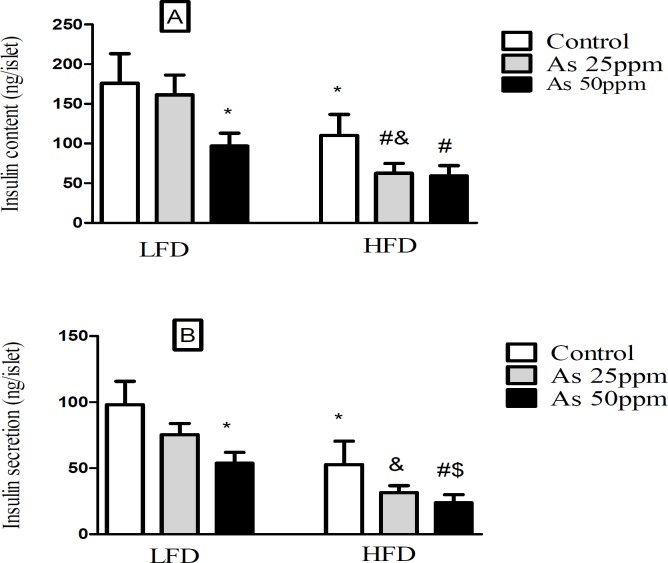
Effect of diet and As exposure on insulin secretion and content of pancreatic islets in control LFD or HFD fed and As 25 or 50 treated LFD or HFD mice (A) Insulin content; (B) Insulin secretion. Values represented as mean ± SD (n = 12, for A-B). *: Significantly different from LFD, #: Significantly different from HFD, &: Significantly different from LFD + As 25 ppm, $: Significantly different from LFD + As 50 ppm. *, #, & and $ *p* < 0.05

**Figure 6:**
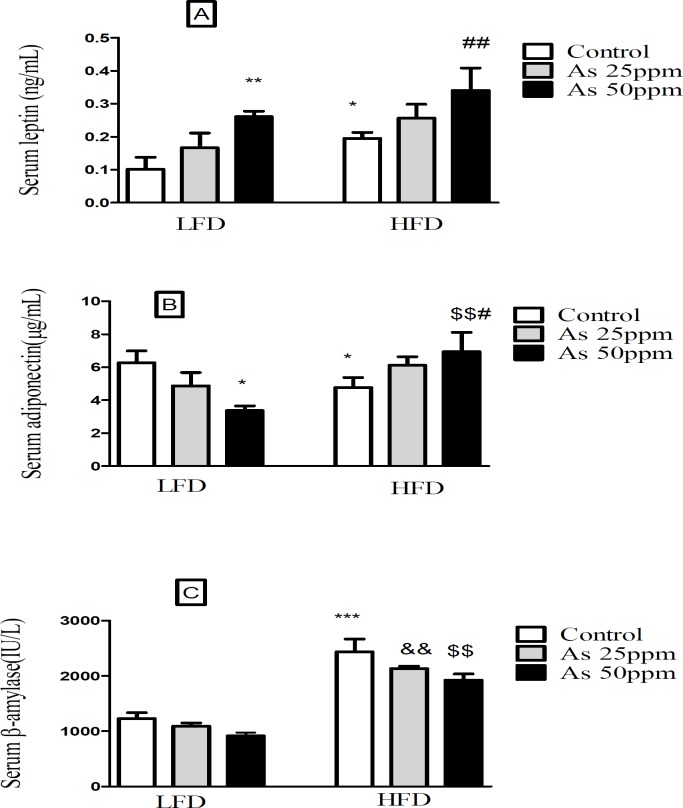
Effects of As and diet on (A) plasma leptin levels; (B) plasma adiponectin levels and (C) plasma β amylase levels in control LFD or HFD fed and As 25 or 50 treated LFD or HFD mice. Values represented as mean ± SD (n = 12, for A-C). *: Significantly different from LFD, #: Significantly different from HFD, &: Significantly different from LFD + As 25 ppm, $: Significantly different from LFD + As 50 ppm. * and # *p <* 0.05, **, ##, && and $$ *p <* 0.01, *** *p <* 0.001

**Figure 7:**
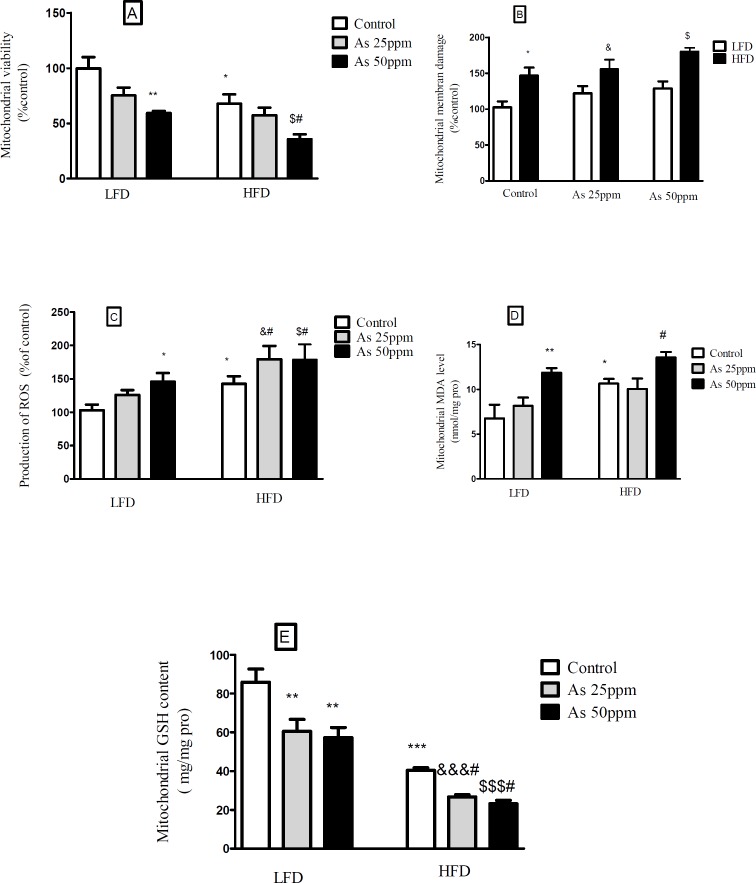
Effects of As and diet on mitochondrial oxidative stress and damage in control LFD or HFD fed and As 25 or 50 treated LFD or HFD mice. (A) Mitochondrial viability; (B) Mitochondrial membrane damage Mitochondrial GSH level; (C) Mitochondrial ROS formation; (D) Mitochondrial MDA level; (E) Mitochondrial GSH level. Values represented as mean ± SD (n = 12, for A-E).*: Significantly different from LFD, #: Significantly different from HFD, &: Significantly different from LFD + As 25 ppm, $: Significantly different from LFD + As 50 ppm. *, #, & and $ *p <* 0.05, ** *p <* 0.01, ***, &&& and $$$ *p <* 0.001

**Figure 8:**
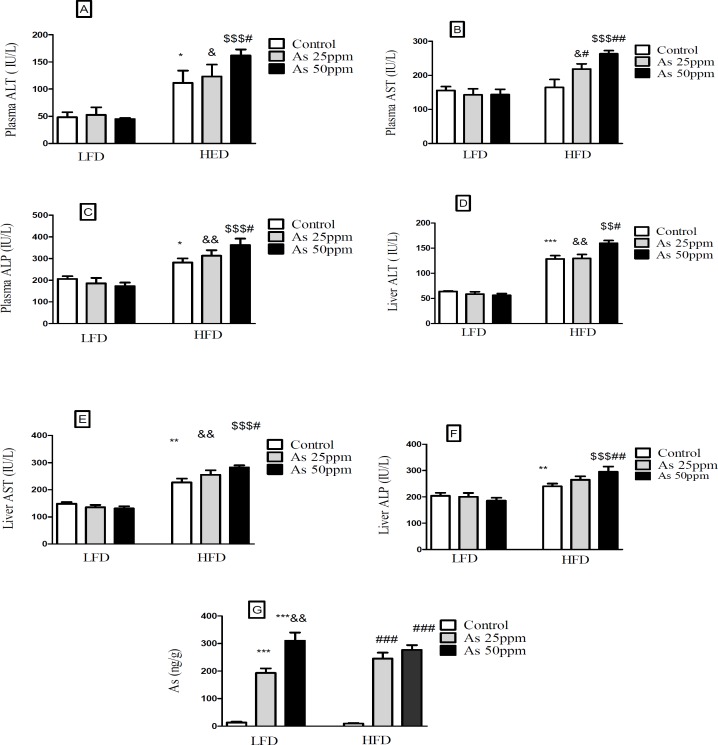
Effects of As and diet on liver and plasma levels of hepatic enzymes and Liver distribution of As in control LFD or HFD fed and As 25 or 50 treated LFD or HFD mice. (A) Plasma ALT; (B) Plasma AST; (C) Plasma ALP; (D) Liver ALT; (E) Liver AST; (F) Liver ALP (G) Liver arsenic distribution. Values represented as mean ± SD (n = 12, for A-E).*: Significantly different from LFD, #: Significantly different from HFD, &: Significantly different from LFD + As 25 ppm, $: Significantly different from LFD + As50 ppm. *, #, and & *p <* 0.05, **, ## and && *p <* 0.01, ***, ### and $$$ *p <* 0.001

**Figure 9:**
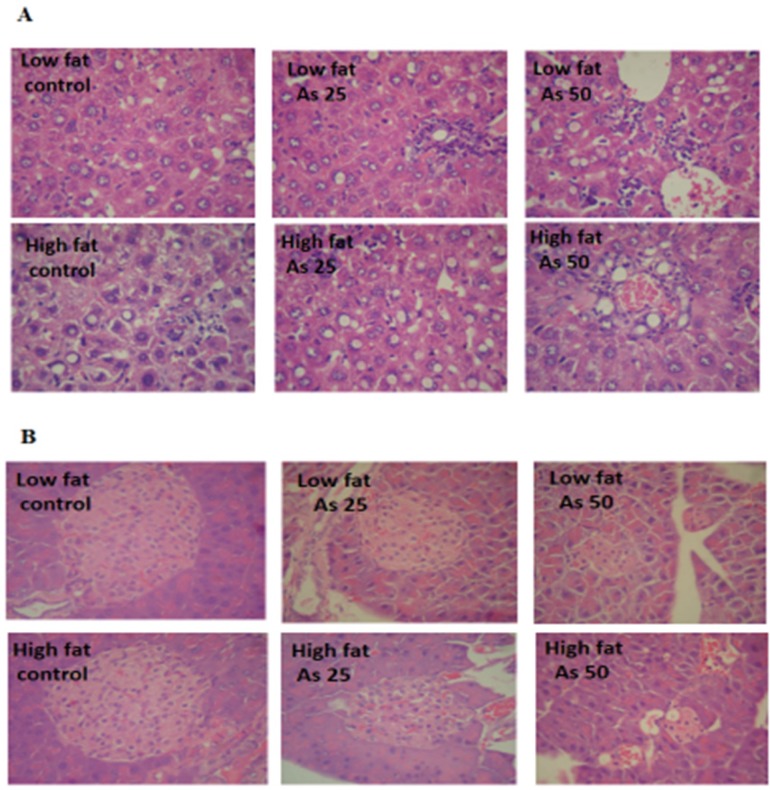
Effects of As and diet on histopathological maps: (A) Pathological maps of liver. (B) Pathological maps of pancreas

The data of average daily arsenic intakes were estimated from water intake. Mice on LFD and HFD exposed to As 25 ppm ingested As 188 and 154.83 μg/day and mice on LFD and HFD exposed to As 50 ppm ingested As 228 and 214 μg/day. These results showed a significant increase of arsenic administration in LFD + As 50 ppm compared to LFD + As 25 ppm 

(*p* < 0.05). Also, the same effect was observed in HFD + As 50 ppm when compared to HFD + As 25 ppm (*p* < 0.05) (Figure 1B).

In general, the mice on LFD consumed more food than the HFD mice. The mice in the control LFD, LFD + As 25 ppm and LFD + As 50 ppm, consumed 10.64 g/day, 10.21 g/day, and 8.68 g/day of food respectively and, control HFD, HFD + As 25 ppm and HFD + As 50 ppm animals fed 9.53 g/day, 8.56 g/day, and 7.63 g/day respectively. Also, the mice in the HFD + As 50 ppm group consumed less food than control LFD (*p* < 0.01) (Figure 1C).

Calorie intakes were estimated using food intake data and caloric densities of LF and HF diets. Although there were no differences between calorie intake in LFD groups, exposure to As showed a significant calorie intake decreases in the HFD groups (*p* < 0.05) 

(57.2 calories/day for control, 51.4 calories/day for As 25 ppm, and 45.8 calories/day for As 50 ppm) (Figure 1D).

*Effect of diet and arsenic exposure on body weight and liver or pancreas weight to body weight ration*

The results in Figure 2A shows body, liver and pancreas weight for LFD and HFD fed control and arsenic treated mice. As expected, control mice fed HFD for 20 weeks weighed more than LFD control mice (*p* < 0.001). Specifically, the HFD consumed mice and exposed to As 25 (35.16 g, *p* < 0.01) and 50 ppm (29.08 g, *p* < 0.001) weighed less than HFD controls (46.33 g). Further, arsenic exposure led to a small weight decrease in LFD fed mice while these differences were not statistically significant. The average total liver to body weight ratio was significantly greater in control HFD mice compared to LFD controls (*p* < 0.001). However, arsenic exposure substantially eliminated the differences in liver to body weight ratio between HFD and LFD mice. Notably, As exposure had no statistically significant effects on liver to body weight ratio in the LFD group, this variable showed an impressive reduction in the HFD-As treated mice (*p* < 0.001) (Figure 2B). 

Further, arsenic exposure decreased the pancreas to body weight ratio in both groups of LFD and HFD mice at 50 ppm (*p* < 0.01) (Figure 2C).

*Effect of diet and arsenic exposure on glucose homeostasis and tolerance*

FBG and OGTT were administered to evaluate glucose homeostasis and tolerance (Figure 3). After 20 week LFD consumption, the control LFD mice showed an average FBG level of 101.83 mg/dL. The HFD feeding for 20 weeks resulted in a statistically significant FBG increase (199.33 mg/dL) in control HFD mice (*p* < 0.001). Moreover, lower FBG levels in HFD mice exposed to 25 and 50 ppm arsenic (127.16 and 114.5 mg/dL respectively) was detected in comparison with control HFD group (*p* < 0.001). The exposure to As showed a tendency to increase FBG in the control LFD (101.83 mg/dL), LFD + As 25 ppm (111 mg/dL), and LFD + As 50 ppm (139.5 mg/dL) groups and these differences were not statistically significant (Figures 3A-B).

Regardless of diet or arsenic exposure, OGTT induced a characteristic rapid rise in blood glucose and peaking within 30 min of glucose challenge. Also, this peaking was followed by a gradual decrease that indicate glucose uptake by the liver and peripheral tissues. Blood glucose levels of LFD fed mice peaked at 353 mg/dL and approached at the baseline levels after 120 min glucose challenge, whereas HFD groups peaked at 428 mg/dL and remained elevated (Figures 3C–E). To quantify glucose tolerance, the area under the curve (AUC) was calculated for all treatment groups. Regardless of arsenic exposure animals, AUC values were significantly higher in HFD fed mice compared with LFD groups (*p* < 0.01). The average AUC values in LFD mice exposed to As 50 ppm (35335 units) were higher than LFD controls (28617 units). However, these differences did not reach at statistical significance. Further, HFD exposed to As 50 ppm increased AUC (46033 units) compared to HFD controls (37032 units) (*p* < 0.01) (Figure 3F).

*Effect of diet and arsenic exposure on insulin resistance*

Fasting plasma insulin (FPI) and insulin tolerance test (ITT) were measured to characterize insulin secretion in response to the glucose challenge and insulin resistance. FPI were affected by HFD and exposure to As. In general, the mice in the HFD groups showed a lower of FPI levels compared with LFD groups (*p* < 0.01). Compared to control HFD mice, FPI values in HFD + As exposure decreased in a concentration dependent manner (*p* < 0.05). Also, LFD + As 50 ppm revealed a significant reduction in FPI when compared to control LFD group (*p* < 0.01) (Figure 4A).

Moreover, the averages of HOMA-IR values were consistently higher in control HFD than LFD group (*p* < 0.05). Arsenic exposure did not change HOMA-IR in LFD fed mice, but it decreased this index in the HFD group in a dose dependent manner (*p* < 0.01 and *p* < 0.001, respectively) (Figure 4B). 

Compared to LFD control mice, the value of HOMA-β was significantly reduced in HFD control group (*p* < 0.05). Further, exposure to As 50 ppm significantly decreased the HOMA-β values in LFD and HFD fed mice compared to their control groups (*p* < 0.05) (Figure 4C).

In the insulin tolerance test (ITT), insulin resistance was carried out in control HFD mice compared with LFD control (*p* < 0.05). Also, arsenic treatment significantly improved the insulin induced blood glucose reduction at 30 min in HFD mice. The ITT-AUC in HFD exposed to As 25 ppm (*p* < 0.05) and As 50 ppm (*p* < 0.01) was significantly lowered than HFD control group (Figures 4D-G).

*Effect of diet and arsenic exposure on insulin secretion and content of pancreatic islets*

Islet’s insulin content and secretion decreased in HFD groups compared with LFD groups (*p* < 0.05). Exposure to As 50 ppm reduced these contents and secretion in LFD and HFD groups compared to their controls (*p* < 0.05). Further, As 25 ppm administration in HFD mice revealed a significant decrease in islets insulin content in comparison with HFD control group (*p* < 0.05) (Figures 5A-B).

*Effect of high fat diet and arsenic on lipid profiles*

Lipid profiles of fasted mice were measured in all treatment groups, and there were no statistical significant between LFD consumed groups, but consumption of HFD was associated with higher plasma TG and VLDL levels (*p* < 0.05). Also, arsenic exposure decreased plasma TG and VLDL in the HFD fed mice compared to the control HFD group in a dose dependent manner (*p* < 0.05). Plasma levels of cholesterol, LDL and HDL were significantly increased by HFD feeding (*p* < 0.01) and, As 50 ppm exposure decreased these variables except HDL in HFD treated mice when compared to control HFD group (*p* < 0.05). Further, the liver TG concentration of control HFD fed mice was higher than control LFD (*p* < 0.001) and, this factor decreased in HFD + As 25 ppm (*p* < 0.05) and HFD + As 50 ppm (*p* < 0.01) when compared to the control HFD group (Table 1).

*Effects of high fat diet and arsenic on the plasma levels of leptin, adiponectin and β amylase*

The present results showed that leptin levels were significantly greater in HFD control mice than LFD control (*p <* 0.05). Further, exposure to As 50 ppm increased plasma leptin level in LFD and HFD consumed animals when compared to their control groups (*p <* 0.01) (Figure 6A). Plasma adiponectin level was significantly lower in control HFD mice compared with LFD control (*p <* 0.05). Although, As 50 ppm exposure could decrease plasma levels of adiponectin in LFD fed mice (*p <* 0.05), this agent increased this variable in the HFD consumed animals (*p <* 0.01) (Figure 6B). The plasma level of β amylase was significantly increased in control HFD mice compared with LFD control (*p <* 0.001). Further, there was a significant difference between HFD + As exposure groups with the same groups in LFD consumed mice (*p <* 0.01), but As administration in LFD and HFD fed mice did not produce impressive changes in the plasma levels of this enzyme compared to their controls (Figure 6C).

*Effects of diet and arsenic exposure on liver mitochondrial viability and damage*

The results showed a significant decrease in the mitochondrial reduction of MTT to formazan in HFD consumed mice compared with LFD (*p <* 0.01). Further, As 50 ppm exposure decreased mitochondrial viability in LFD (*p <* 0.01) and HFD (*p <* 0.05) fed groups in comparison to their controls (Figure 7A). To uptake of cationic fluorescent dye, rhodamine 123 has been used for the measurement of mitochondrial membrane potential collapse. As shown in Figure 7B, HFD significantly induced MMP collapse in isolated liver mitochondria (*p <* 0.05), but asernic exposure had no statistically significant effects in both LFD and HFD fed mice when compared to their controls.

*Effects of diet and arsenic exposure on liver mitochondrial oxidative stress*

The liver was chosen as a target organ to confirm the oxidative stress based on pathological and mitochondrial analyses. Increased ROS formation is expressed as DCF fluorescence intensity unit. As shown in Figure 7C, HFD induced a significant rise at ROS formation in liver’s mitochondria (*p <* 0.05). Exposure to As 50 ppm increased this variable in LFD fed mice (*p <* 0.05) when compared to its control. Also, compared to the control HFD group, over generation of ROS has been occurred in HFD As 25 and 50 ppm treated mice (*p <* 0.05). The results of lipid peroxidation revealed that mitochondrial MDA level was significantly higher in the control HFD mice compared with LFD group (*p <* 0.05). Also, As 50 ppm exposure significantly increased this variable in LFD (*p <* 0.01) and HFD (*p <* 0.05) fed mice when compared to their controls (Figure 7D).

Glutathione assessment results showed a significant decrease in the control HFD group compared to the control LFD fed mice (*p <* 0.001). Further, two doses of arsenic administration decreased more this antioxidant enzyme in LFD (*p <* 0.01) and HFD (*p <* 0.05) consumed animals compared to their control groups (Figure 7E).

*Effect of high fat diet and arsenic on liver and plasma levels of hepatic enzymes*

Hepatic enzymes measurement indicated that plasma levels of ALT and ALP increased in the control HFD compared to LFD control group (*p <* 0.05). Further, exposure to As 50 ppm increased these enzymes levels in HFD consumed animals in comparison with the control HFD group (*p <* 0.05). Also, a significant plasma AST level elevation was observed after As 25 ppm (*p <* 0.05) and 50 ppm (*p <* 0.01) administration in HFD fed mice compared to the control HFD group (Figures 8A-C). Liver assessment revealed that, alone HFD consumption was associated with the higher levels of AST (*p <* 0.01), ALT (*p <* 0.001) and ALP (*p <* 0.01) compared to LFD control group (*p <* 0.05). Further, exposure to As 50 ppm increased more hepatic enzymes such as AST (*p <* 0.05), ALT (*p <* 0.05) and ALP (*p <* 0.01) in HFD consumed mice compared to the control HFD group(Figures 8D-F).

*Liver distribution of arsenic*

Exposure to As 25 and 50 ppm resulted in accumulation of this agent in the liver of LFD and HFD treated mice (*p <* 0.001). Also, there was a significant increase of liver’s arsenic accumulation in As 50 ppm compared to As 25 ppm (*p* < 0.01) (Figure 8G).

*Histopathological Analysis*

Liver lobular structures in the control LFD was clear and regular, and single layer of hepatocytes arranged around the central vein in a radial Pattern under the light microscope. However, a few numbers of hepatocytes showed fat deposit (Figure 9A). In LFD consumed As 25 and As 50 groups, fatty changes were increased in hepatocytes. Infiltration of inflammatory cells in interstitial tissue of the livers of these animals was also observed. In control HFD, fatty changes were higher than in the control LFD fed mice. In HFD exposed to As 25 and As 50 animals, fatty change and infiltration of inflammatory cells were more than control HFD group. In addition, congestion of red blood cells was observed in HFD As 50 treated animal.

Exocrine and endocrine portion of the all pancreases revealed a normal appearance in control and experimental groups. However, in all arsenic treated groups, the diameter of islets were significantly decreased in a dose dependent manner (*p <* 0.05). These results are shown in Figure 9 and Table 2.

## Discussion

The interaction between diet and environment plays an important role in human diseases (16). Diabetogenic effects of HFD and arsenic consumptions have been determined in previous study. Hence, the present study examined the combination of arsenic exposure and obesity to evaluate the diabetogenic mechanism of them. The results indicated that HFD decreased water intake and administration of arsenic revealed the same effect in LFD and HFD groups in a dose dependent manner. Although animals that treated with As 50 ppm drank less water, they showed a significant arsenic intake. Also, concomitant As and HFD administration produced food and calorie intake limitation, body weight, liver and pancreas weight to body weight ratio reduction and, these effects were more evident in As 50 ppm treated mice. These results are in agreement with Paul *et al.* study (16). Some studies suggested that HFD feeding developed insulin resistance concomitant with high blood glucose levels. Body’s resistance to insulin and falling insulin production of pancreatic β cells are two main factors in HFD induced type 2 diabetes (33). The exact relationships between high fat diet, insulin resistance, and type 2 diabetes are pathological accumulation roles of fatty acids or fatty acid derivatives such as polyunsaturated fat in muscle or liver, that produced impairment of insulin sensitivity (34, 35). OGTT is a simple test that reflected body efficiency to dispose glucose after oral glucose load. This test revealed the glucose and insulin dynamics of physiological conditions similar to glucose clamp or insulin sensitivity test. Impaired glucose tolerance is reflected through the increase of plasma glucose disappearance curve (AUC) (36). 

Recent evidence indicates a relationship between arsenic exposure and prevalence of type 2 diabetes. Further, drinking water containing high dose of inorganic arsenic can synergistically act with high fat diet to produce impairing glucose tolerance in mice (37). However, typical symptoms of type 2 diabetes are insulin resistance and hyperinsulinemia, but arsenic related diabetes was not associated with them Arsenic revealed a potent inhibitor of insulin stimulated glucose uptake and glucose stimulated β-cells insulin secretion. Thus, these two mechanisms can induce fasting hyperglycemia and impaired OGTT, decreased FPI, and HOMA-IR (38). The present results showed that although HFD consumption increased FBG, HOMA-IR, and ITT AUC, arsenic administration reduced all of them. Further, not only HFD induced hypoinsulinemia and decreased HOMA-β but also arsenic treated revealed more reduction in these variables. It was established that hypoglycemia associated with glycogenolysis has been occurred through the short-term arsenic utilization in rats (39). Further, pyruvate dehydrogenase was considered an important molecular target of arsenic that produce hypoglycemia through the gluconeogenesis reduction (40). Hence, it could be suggested that chronic arsenic administration showed the same effect on FBG as well as short-term. But, the underlying mechanism remains unknown which requires further research. 

The results of OGTT AUC indicated that HFD could enhance glucose tolerance in mice and arsenic consumption aggravate that in a concentration dependent manner. Hence, as shown in the previous study, the present data suggest that exposure to HFD consumption and As may induced diabetes differ from type 2 diabetes which is characterized by fasting hyperglycemia and impaired glucose tolerance.

Further, after islet isolation and insulin secretion measurement, it was revealed that high dose of arsenic could reduce insulin content and secretion from the isolated islet and these effects were more evident in HFD consumed mice. Pancreatic β-cells are more susceptible to oxidative stress because its antioxidant defense is weak. One research suggested that HFD induced chronic oxidative stress and oxidative damage which cause to β-cell dysfunction (41). Arsenic produces the impairment of glucose stimulated insulin secretion in β-cells at low and high concentrations through the oxidative stress and over generation of ROS (42). Hence, the present study suggests that one of the involved mechanisms in the reduction of glucose-stimulated insulin secretion is induction of excessive oxidative stress by HFD and arsenic animal’s consumption.

HDF can result in dyslipidemic changes such as increasing serum levels of TG, total cholesterol, LDL and VLDL. Further, this diet increased TG content of the liver due to the excess influx of fatty acids into the liver. Moreover, increased production of ROS as well as reduced antioxidant defense mechanisms have been suggested playing a role in both dyslipidemia induced by HFD consumption (43). Hence, in accordance with previous study, the present data indicate that HFD induced hyperlipidemia through the increase lipid profiles such as TG, cholesterol, LDL, HDL in the liver and plasma, but As consumption revealed an impressive reduction in them except HDL and, it can be suggested that HFD feeding induced dyslipidemic variation via oxidative stress and imbalance between ROS and antioxidant enzymes activities. Previous study has shown that arsenic inhibits the responsible signal transduction mechanisms for adipocytes differentiation. This differentiation is responsible for fat TG accumulation in adipose tissues. Thus, the limited fat accumulation in HFD mice exposed to arsenic is due to the inhibition of adipocytes differentiation by arsenic (16). Also, the obtained results indicated that although arsenic utilization could reverse the effects of HFD on increasing β amylase level and decreasing in plasma adiponectin, this chemical element revealed a similar effect of HFD and LFD consumption on plasma leptin level enhancement. In the conform of present results, one study showed that HFD induced an increase in serum leptin and decreased in adiponectin levels and suggested that this diet impairs glucose control via increasing leptin secretion (44). Alterations in adipokines secretion such as leptin and adiponectin could play an important role in the diet-induced diabetes. Plasma leptin concentration acts to regulate the function of muscle cells and pancreatic β-cells. Further, hyperleptinemia may cause to obesity-related complications including development of type 2 diabetes. New research reveals an association between arsenic exposure and leptin (45). Obesity concomitant with arsenic exposed mice showed a remarkable leptin resistance and higher circulating levels of leptin that is in agreement with our research (46). Adiponectin synthesized in adipose tissue and regulate glucose and lipid metabolism by targeting the skeletal muscle and liver. Hypoadiponectinemia has an important role in obesity related insulin resistance, inflammation, type 2 diabetes, and metabolic syndrome (44). Further, there is a negative correlation between adiponectin and obesity or lipid profile such as TG, cholesterol, LDL-c and VLDL. The main involved mechanisms of adiponectin in lipid oxidation are the regulation of produce or action of TG-related metabolism proteins such as acyl CoA oxidase, activated protein kinase, and peroxisome proliferator activated receptorγ (47). Therefore, hypolipidemic activity of arsenic administration in the present study may occur through the enhancement of plasma adiponectin levels and its lipid oxidation mechanisms. Serum amylase is one of the main variables for pancreatic functional determination. This enzyme levels increase through the pancreatic cells injury. Further, elevation of serum amylase level has been occurred in HFD fed animals due to pancreatic cells damage which, is in agreement of the present HFD consumption results in the mice (48). Consistent with the oxidative stress, the reduction average of α and β amylase activity was observed by arsenic administration in rice (49). Also, there is no animal’s research about the effects of arsenic on β amylase level. Hence, present finding suggest that arsenic has a tendency to decrease plasma β amylase level via enhancement of oxidative stress, but more studies are necessary to reveal the main mechanisms of this event. 

The results of isolated liver mitochondria assessment in this research revealed that, arsenic could induce oxidative stress through the ROS overproduction, increase MDA concentration and decrease GSH in all animals, and these effects were more evident in HFD consumed mice. HFD and arsenic consumption could reduce viability of liver mitochondria in HFD + As-treated mice. Moreover, mitochondrial damage has been occurred in HFD concomitant with arsenic administration animals in a dose dependent manner. Hepatotoxicity has been occurred through the elevation of plasma and liver hepatic enzymes in HFD consumed mice and after arsenic administration the worst situation was observed in them.

It is well known that ROS and MDA are oxidative stress bio markers. Exposure to As with HFD leads to an oxidative stress dependent on production of ROS and lipid peroxidation of membranes. Glutathione has been shown as important biomarkers of oxidative stress, which forms the first line of antioxidant defense against arsenic induced damages, and a reduction in tissue GSH level indicated to oxidative damage (50). So, it could be suggested that animals co-treatment with arsenic and HFD aggravate liver mitochondrial damage and viability through the oxidative stress and reduction of GSH level.

The liver is an essential tissue that performed a wide range of biochemical, metabolic, and drug metabolites (51). AST, ALT, and ALP enzymes are livers biomarker associated with liver dysfunction or damage (52). The cytosolic leakage of AST and ALT has been occurred into the blood stream through the liver parenchymal injury, and increase the plasma levels of these enzymes. Some research showed a significant hepatic enzymes enhancement in HFD and arsenic treated rats (50). So, in accordance with previous studies it could be suggested that HFD and arsenic administration induced hepatotoxicity via increase oxidative stress and plasma or liver levels of ALT, AST, and ALP. 

In histological assessments it was found that the appearance of inflammatory cells in liver tissue suggests that the As can interact with proteins and enzymes in the interstitial tissue of the liver, interfering with the antioxidant defense mechanism and leading to generation of ROS, which in turn may induce an inflammatory response (53). Also, the diameter of islets was markedly decreased in arsenic treated animals which may relate to decrease insulin secretion.

In conclusion, the present results indicate that HFD and As concomitant administration induced differ form of type 2 diabetes, which is characterized by impaired glucose tolerance and islet’s insulin secretion or content without typical symptoms of type 2 diabetes such as insulin resistance, hyperglycemia, and hyperinsulinemia. Also, failure of the pancreatic β cells, hepatotoxicity, hyperleptinemia, hypolipidemia, hypoadiponectinemia and liver oxidative stress and damage have been occurred in this model of diabetes. But, further researches are required to clarify the other mechanisms of these events. 
